# CT facilitates improved diagnosis of adult intestinal malrotation: a 7-year retrospective study based on 332 cases

**DOI:** 10.1186/s13244-021-00999-3

**Published:** 2021-04-30

**Authors:** Ziman Xiong, Yaqi Shen, John N. Morelli, Zhen Li, Xuemei Hu, Daoyu Hu

**Affiliations:** 1 Department of Radiology, Tongji Hospital, Tongji Medical College, Huazhong University of Science and Technology, 1095 Jiefang Avenue, Qiaokou District, Wuhan, 430030 Hubei China; 2 Department of Radiology, St. John’s Medical Center, Tulsa, OK USA

**Keywords:** Intestinal malrotation, Adults, Computed tomography, Diagnosis

## Abstract

**Objective:**

To classify adult intestinal malrotation by CT.

**Methods:**

This retrospective study enrolled adults diagnosed with intestinal malrotation who underwent abdominal CT at our institution between June 1, 2013, and August 30, 2020. All patients’ clinical information was recorded. Patients were divided into groups undergoing surgical and conservative management. The duodenum (nonrotation, partial rotation, and malrotation), jejunum, cecum, and the superior mesenteric artery/superior mesenteric vein relationship were reviewed on the CT images of each patient, and classification criteria developed based on the first three items. For each patient, each item was assessed separately by three radiologists. Consensus was required from at least two of them.

**Results:**

A total of 332 eligible patients (218 men and 114 women; mean age 51.0 ± 15.3 years) were ultimately included and classified into ten types of malrotation. Duodenal partial rotation was present in most (73.2%, 243/332) with only 25% (83/332) demonstrating nonrotation. The jejunum was located in the right abdomen in 98.2% (326/332) of cases, and an ectopic cecum was found in only 12% (40/332, 29 cases with a left cecum, 7 pelvic, and 4 at midline). Asymptomatic patients comprised 56.6% (188/332) of cases, much higher than that in previous studies (17%, *n* = 82, *p* < .001), comprised mainly of patients with duodenal partial rotation (80.3%, 151/188). In 91 patients with detailed clinical data available (12 managed surgically and 79 conservatively), a significant difference in malrotation CT categorization was identified (*p* = .016).

**Conclusions:**

CT enables greater detection of asymptomatic intestinal malrotation, enabling classification into multiple potentially clinically relevant subtypes.

**Supplementary Information:**

The online version contains supplementary material available at 10.1186/s13244-021-00999-3.

## Key points

- CT allows detection asymptomatic intestinal malrotation.
- Difference was observed in CT classification between the surgical and conservative groups.
- Classification of intestinal malrotation enables identification of high-risk patients.

## Introduction

Intestinal malrotation is a congenital anomaly, often considered a pediatric disorder since most patients are diagnosed within the first year of life [1]. Definitive diagnosis can be made by typical symptoms of bilious vomiting in combination with an abnormal upper gastrointestinal (UGI) series [2–4]. In adult patients, however, the diagnosis is often incidental. Because of atypical symptoms, these patients are often initially diagnosed with other diseases [5] with further imaging studies or surgery revealing intestinal abnormalities. In fact, the number of adult malrotation patients is far underestimated. Nehra et al. [6] found adults accounting for 48% (82/170) of malrotation cases with 17% of these asymptomatic. However, being asymptomatic at a given point in time does not guarantee a patient will remain asymptomatic for life. In some cases, coexisting diseases or changes in physical condition could trigger a series of complications, including life-threatening volvulus [7, 8]. There are a lack of research on asymptomatic patients and still no clear indication which patients may develop dangerous complications in the future requiring surgery [9, 10].

The UGI series remains the current gold standard for the diagnosis of intestinal malrotation and is widely used in pediatrics [11]. In addition, ultrasound (US) is also recommended for the screening of malrotation with volvulus [12]. Some studies have been conducted in pediatric patients using UGI or US [13–16]. However, in adult patients, computed tomography (CT) scans, especially with intravenous and oral contrast, have greater diagnostic value, and some studies recommend CT as the first choice in adult patients with suspected malrotation [5, 8, 17]. CT not only displays abnormal signs visible on UGI and US, but also avoids the influence of intestinal gas, enabling acquisition of complete anatomical information regarding the bowel. There have also been studies using CT to classify intestinal variation in adult patients [18, 19], but these included fewer than 20 patients. In addition, the literature on adult intestinal malrotation mostly consists of case reports.

In order to improve the awareness of radiologists and clinicians about adult intestinal malrotation, in the current study, records from all adult patients diagnosed with intestinal malrotation at our institution were reviewed since 2013 and analysis of relevant clinical data as well as CT images performed.

## Materials and methods

### Subjects

This retrospective study was approved by the local institutional review board, and informed consent was waived. The electronic medical record system in our institution between June 1, 2013, and August 30, 2020, was reviewed using “intestinal malrotation” and “midgut malrotation” as keywords for searching. A series of inclusion/exclusion criteria were constructed to select eligible cases followed by further analysis.

Criteria for selecting the subjects were as follows: (a) adult patients (age ≥ 18 years old); (b) intestinal malrotation confirmed by surgery or by consensus among three radiologists (Additional file 1: Methods); (c) abdominal CT images available; and (d) clinical data available (at least sex, age, and symptoms at initial diagnosis). Exclusion criteria were: (a) CT images unavailable or the scan not including the entire abdomen; (b) CT scan performed after small bowel surgery; (c) clinical data unavailable; and (d) malrotation ruled out after review by a senior radiologist.

Clinical data of patients meeting the above criteria were reviewed (sex, age, symptoms at initial diagnosis, duration of symptoms, comorbidities, and treatment), and the patients were divided into groups based on whether they underwent surgical or conservative management. Further analysis of these two groups was then performed.

### Image acquisition

All patients underwent abdominal CT at our institution. Vendors of CT equipment included GE Healthcare (Lightspeed 16, Lightspeed 64, Discovery 750, and Brightspeed Elite, Waukesha, WI, USA), Philips Healthcare (Brilliance 16 and ICT256, Amsterdam, the Netherlands), and Toshiba Medical Systems (Aquilion One, Otawara, Japan). The CT tube voltage was 120 or 140 kVp, and the automatically controlled tube current was 110–720 mAs. The CT scan covered from the diaphragm to the pubic symphysis. If possible, fast for 4-6 hours before the scan, followed by ingestion of 1000–1500 ml of 2.5% mannitol water solution orally 40–60 min prior to the scan. Some patients underwent an unenhanced CT, followed by contrast-enhanced imaging; others were scanned with IV contrast only. Contrast-enhanced CT was performed after a rapid bolus of iopromide (Ultravist 370, 370 mg/mL, Bayer Schering Pharma, Berlin, Germany) (1.5 mL/kg) at a rate of 3–5 mL/s, followed by a 20-mL saline flush using a power injector. Images were routinely obtained in the arterial, intestinal, or portal venous phases at 30, 45, or 70 s, respectively.

### Image interpretation

CT images of all patients were evaluated separately by three radiologists. For each patient, at least two radiologists had to agree on each item of evaluation. The assessment included:

1. Duodenum crossing the midline (the space between the aorta and the superior mesenteric artery (SMA)) or not (a duodenum failing to cross the midline was considered duodenal nonrotation, a duodenum crossing midline then suddenly returning to the right of midline was considered duodenal partial rotation, and a duodenum crossing midline without folding back was considered duodenal malrotation),
2. The main site of the jejunum,
3. The location of the cecum,
4. Position of SMA relative to superior mesenteric vein (SMV),
5. The presence of the whirlpool sign.

**Fig. 1:**
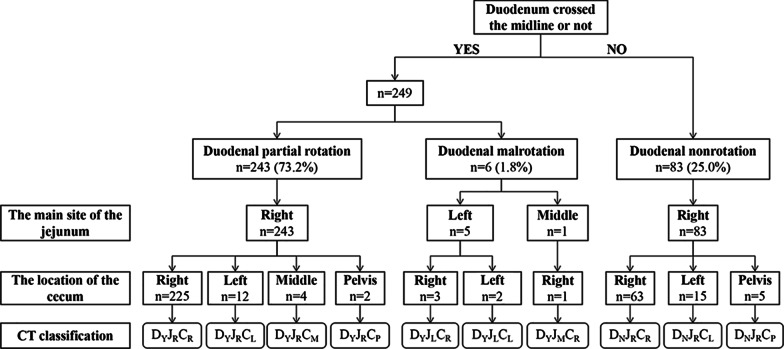
Process and results of classification of abdominal CT images

CT classification of patients was based on (1)–(3) (Fig. 1).

### Statistical analysis

All statistical analysis was performed with SPSS 26.0. To compare results with those of previous studies and to compare variables between the two groups herein, the independent Student t-test was used for continuous variables and Chi-square or Fisher’s exact test for categorical variables. All analyses were considered significant at *p* values of less than 0.05 (two-tailed).

## Results

A final 332 eligible adult patients with intestinal malrotation were included in the study. Since 188 of these patients were incidentally identified and did not exhibit symptoms related to intestinal malrotation or receive relevant treatment, they were included in the asymptomatic group, and the course of their treatment was not further documented. Of the 144 patients whose symptoms were recorded, 8 patients had comorbidities that may be associated with symptoms (gastroptosis, liver cysts, irritable bowel syndrome, ileocecal tumors, rectal cancer). Since the possibility that the symptoms were caused by malrotation could not be ruled out, these symptoms were still recorded. In addition, treatment information was not available for 53 outpatients. Therefore, detailed clinical and treatment information was available for 91 patients, who were categorized into surgical and conservative management groups for further analysis (Additional file 1: Fig. S1).

### Clinical characteristics of all cases

The clinical characteristics of all cases are summarized in Table 1.

**Table 1:** Clinical characteristics of 332 adult malrotation patients

Parameters	Total (*n* = 332) No. (%)
Gender ratio (male/female)	1.91:1
Age, years	
Mean	51.0 ± 15.3
Range	18–97
Asymptomatic patients	188 (56.6)
Symptoms at diagnosis^†^ (*n* = 144)	
Abdominal pain	114 (79.2)
Bloating	37 (25.7)
Vomiting	18 (12.5)
Nausea	7 (4.9)
Diarrhea	6 (4.2)
Constipation	4 (2.8)
Complication: volvulus	7 (2.1)
Duration of symptoms^#^ (*n* = 91)	
Hours/days	25 (27.5)
Weeks	16 (17.6)
Months	24 (26.4)
Years	26 (28.5)
Treatment^#^ (*n* = 91)	
Conservative treatment	79 (86.8)
Surgical treatment	12 (13.2)

^†^Patients’ first symptoms are duplicated (coexistence of several symptoms)

^#^Duration of symptoms and treatment modalities were obtained from 91 patients, and outpatients without detailed records and asymptomatic patients with predominantly other conditions (kidney stones, cirrhosis, hypertension, etc.) were not recorded

An overall male predominance in both groups was observed (male-to-female ratios were: overall 1.91:1, surgical group 2:1, and conservative group 1.47:1) and the age range of included patients was broad (range 18–97). The proportion of asymptomatic patients in the current cohort was 56.6% (188/332), while the proportion of patients with volvulus was 2.1% (7/332). Abdominal pain was still the main complaint among all patients, and chronic symptoms (complaints lasting over one month) were observed in more than half of patients (*n* = 91, 54.9%) with some patients experiencing symptoms for years or even decades. The proportion of patients undergoing surgery was 13.2% (12/91).

### CT findings of all cases

The CT findings of all patients are summarized in Table 2.

**Table 2:** CT findings of 332 adult malrotation patients

	Total (*n* = 332) No. (%)
*CT classification*	
Duodenal partial rotation	
D_Y_J_R_C_R_	225 (67.8)
D_Y_J_R_C_L_	12 (3.6)
D_Y_J_R_C_M_	4 (1.2)
D_Y_J_R_C_P_	2 (0.6)
Duodenal nonrotation	
D_N_J_R_C_R_	63 (19.0)
D_N_J_R_C_L_	15 (4.5)
D_N_J_R_C_P_	5 (1.5)
Duodenal rotation	
D_Y_J_M_C_R_	1 (0.3)
D_Y_J_L_C_R_	3 (0.9)
D_Y_J_L_C_L_	2 (0.6)
*Position of SMA relative to SMV*	
Left rear	165 (49.7)
Left	73 (22.0)
Left front	66 (19.9)
Right rear	28 (8.4)
Whirlpool sign	11(3.3)

*SMA* superior mesenteric artery, *SMV* superior mesenteric vein

**Fig. 2:**
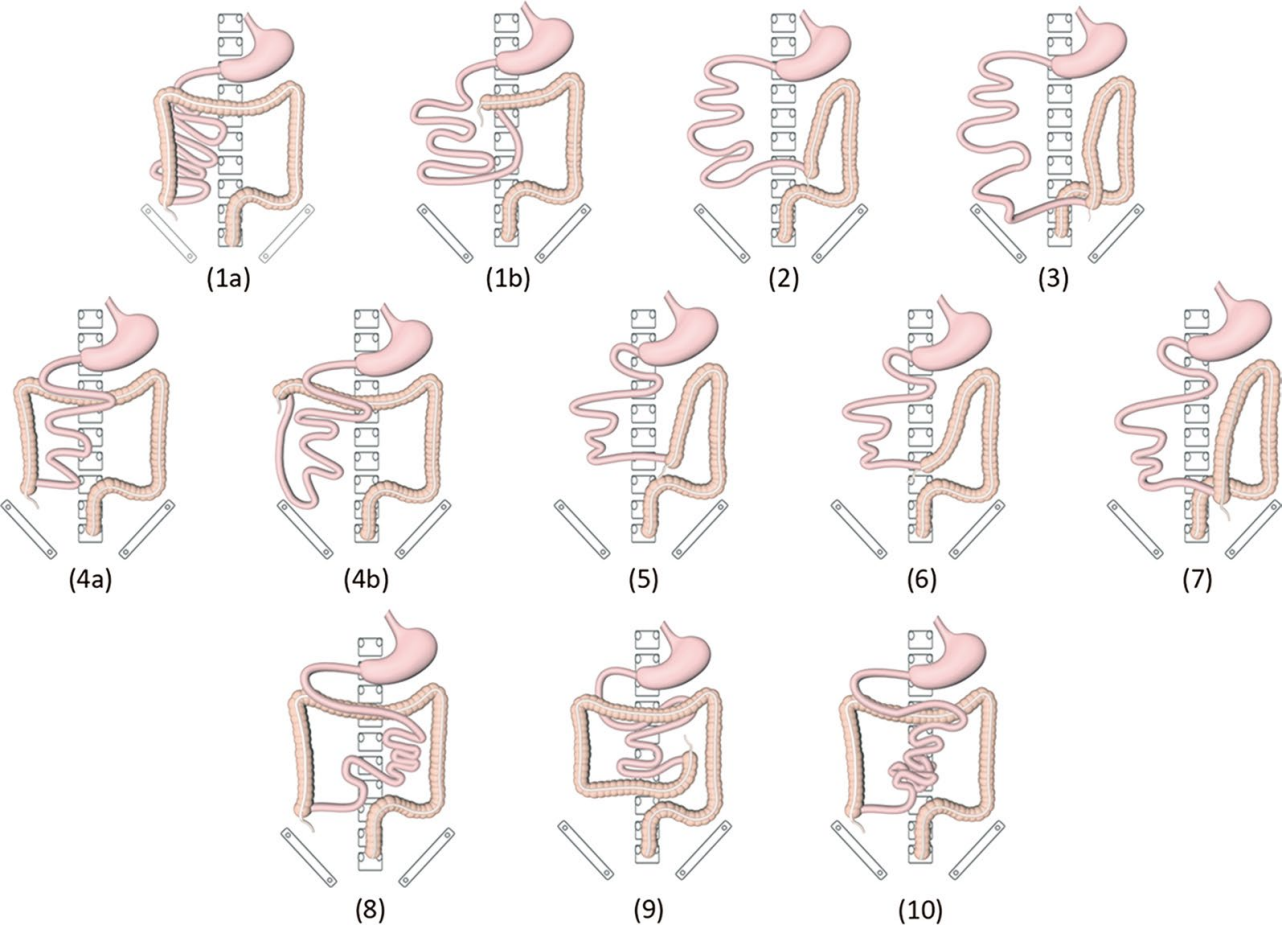
Schematic diagram of the ten subtypes. (**1a**) and (**1b**) two cases of D_N_J_R_C_R_ subtype: normal and high cecum; (**2**) D_N_J_R_C_L_; (**3**) D_N_J_R_C_P_; (**4a**) and (**4b**) two cases of D_Y_J_R_C_R_ subtype: normal and high cecum; (**5**) D_Y_J_R_C_L_; (**6**) D_Y_J_R_C_M_; (**7**) D_Y_J_R_C_P_; (**8**) D_Y_J_L_C_R_; (**9**) D_Y_J_L_C_L_; (**10**) D_Y_J_M_C_R_

**Fig. 3:**
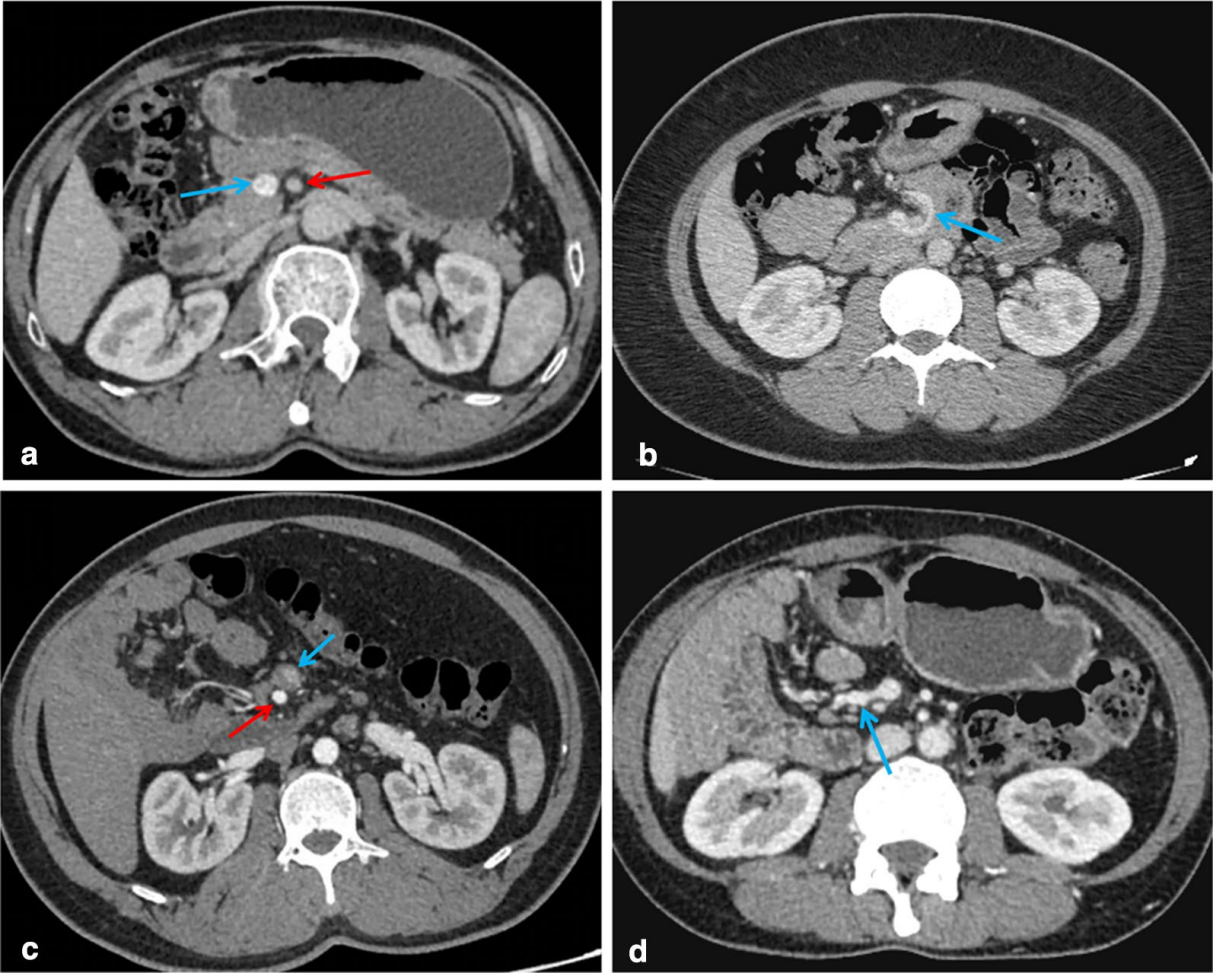
Relationship between the superior mesenteric artery (red arrow) and superior mesenteric vein (blue arrow). **a** Normal (SMA on the left side of SMV); **b** the SMV forms an angle around the SMA toward the right abdominal cavity; **c** abnormal (inversion); 3d: the SMV goes straight to the right abdomen

**Fig. 4:**
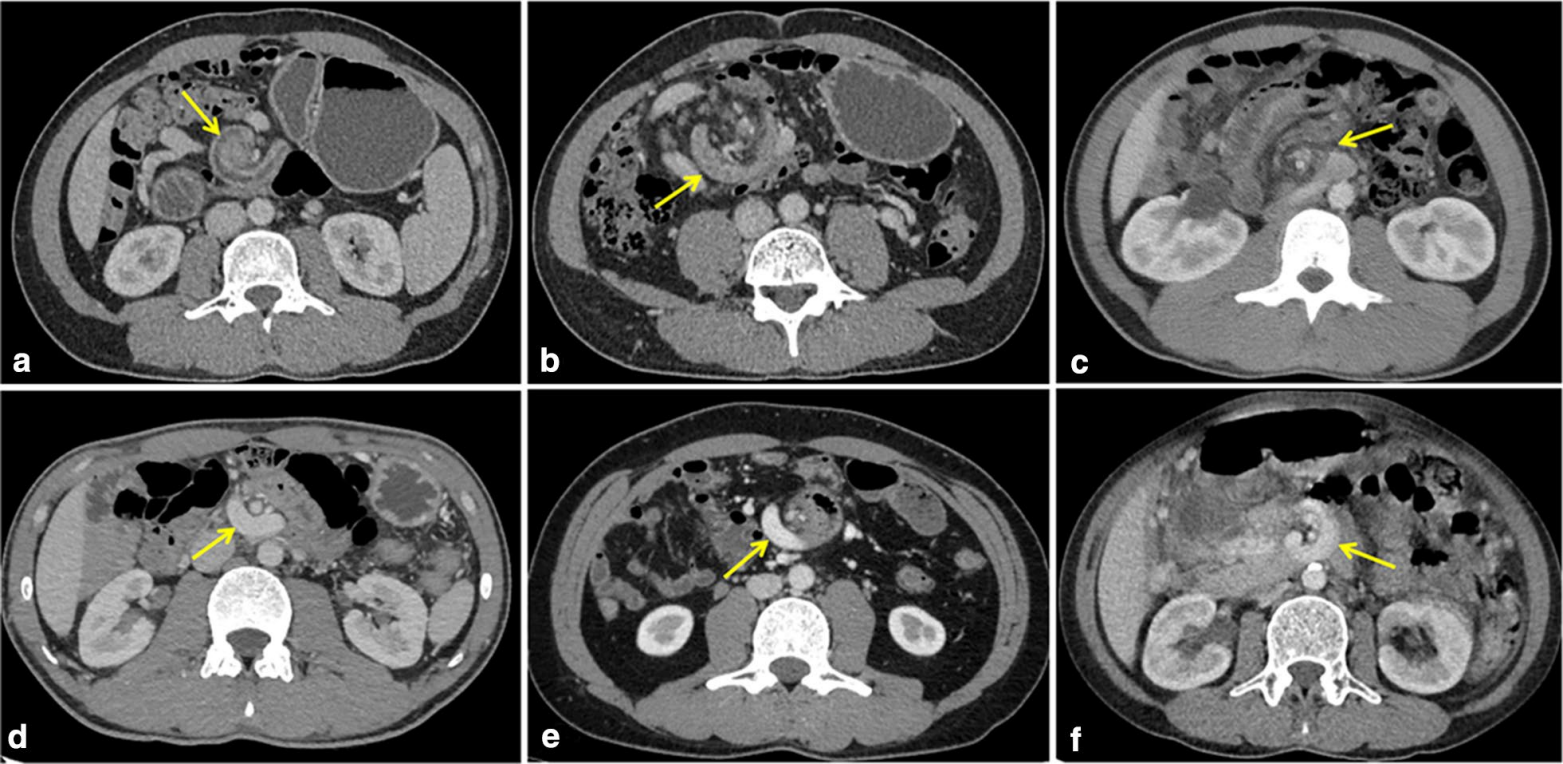
“Whirlpool sign” in different patients. **a**, **b** A 34-year-old male with volvulus from the surgical group (D_Y_J_R_C_R_, type 4a). The images show torsion of duodenum, proximal jejunum and mesenteric vessels (arrow). **c** A 30-year-old male with volvulus from the surgical group (D_Y_J_R_C_M_, type 6). **d**, **e** Two males with volvulus from the conservative group (D_Y_J_R_C_R_, type 4a). The degree of torsion of vessels and intestines is mild (arrow). **f** A 51-year-old asymptomatic female (D_Y_J_R_C_R_, type 4a). The portal vein rotates anterior to the superior mesenteric artery

CT images of 332 patients were categorized into ten types of malrotation based on the orientation of the duodenum, jejunum, and cecum (Fig. 2). The majority of patients showed duodenal partial rotation (73.2%, 243/332) (4a–b, 5, 6, and 7 in Fig. 2) and only 25.0% (83/332) duodenal nonrotation (1a–b, 2, and 3 in Fig. 2). In both scenarios, the jejunum was typically located in the right abdomen. For the remaining six patients who exhibited duodenal malrotation, the duodenum crossed the midline and did not fold back [D_Y_J_L_C_R_ (Fig. 2, type 8), D_Y_J_L_C_L_ (Fig. 2, type 9), D_Y_J_M_C_R_ (Fig. 2, type 10)]; however, the anatomy in such cases was still abnormal. Overall, most patients exhibited a normal SMA/SMV relationship (91.6%, 304/332). Among these, SMV angulation followed by a right-sided deviation was most common (98.7%, 300/304), whereas a straight inferior course was noted in four cases (D_Y_J_L_C_R_, D_Y_J_M_C_R_). However, SMV angulation was not seen in patients with an abnormal SMA/SMV relationship, all of whom showed SMV coursing directly to the right or left abdomen (Fig. 3). In addition, the “whirlpool sign” was found in 11 patients, including six cases with volvulus, four asymptomatic cases, and one outpatient (Fig. 4).

### Comparison of the surgical and conservative groups

The clinical and CT characteristics of surgically and conservatively managed groups are shown in Table 3.

**Table 3:** Clinical and CT characteristics of surgical and conservative groups

Parameters	Surgical group (*n* = 12)	Conservative group (*n* = 79)	*p*
Gender ratio (male/female)	2:1	1.47:1	.76
Age, years			
Mean	50.4 ± 17.5	51.2 ± 18.0	.89
Range	26–73	18–84	
Symptoms			
Abdominal pain	11 (91.7)	63 (79.7)	.45
Bloating	7 (58.3)	19 (24.1)	
Vomiting	3 (25.0)	14 (17.7)	
Nausea	3 (25.0)	3 (3.8)	
Diarrhea	–	5 (6.3)	
Constipation	–	2 (2.5)	
Intestinal obstruction (*n* = 19)	7 (58.3)	12 (15.2)	.002
Complication: volvulus (*n* = 7)	3 (25.0)	4 (5.1)	.046
Duration of symptoms			.36
Hours/days	6 (50.0)	19 (24.1)	
Weeks	2 (16.7)	14 (17.7)	
Months	2 (16.7)	22 (27.8)	
Years	2 (16.7)	24 (30.4)	
*CT classification*^‡^			.016
Duodenal partial rotation			.33
D_Y_J_R_C_R_	3 (25.0)	51 (64.5)	.013
D_Y_J_R_C_L_	1 (8.3)	1 (1.3)	
D_Y_J_R_C_M_	1 (8.3)	1 (1.3)	
D_Y_J_R_C_P_	1 (8.3)	–	
Duodenal nonrotation			.52
D_N_J_R_C_R_	5 (41.7)	20 (25.3)	
D_N_J_R_C_L_	–	2 (2.5)	
D_N_J_R_C_P_	–	1 (1.3)	
Duodenal rotation			.44
D_Y_J_L_C_R_	1 (8.3)	1 (1.3)	
D_Y_J_L_C_L_	–	2 (2.5)	
*Position of SMA relative to SMV*			.75
Left rear	7	34	
Left	3	27	
Left front	2	12	
Right rear	0	6	
Whirlpool sign	2	4	

*SMA* superior mesenteric artery, *SMV* superior mesenteric vein

^‡^Fisher’s exact test was used to determine significance of the difference in CT classification between the two groups (*p* = .016 < 0.05)

**Fig. 5:**
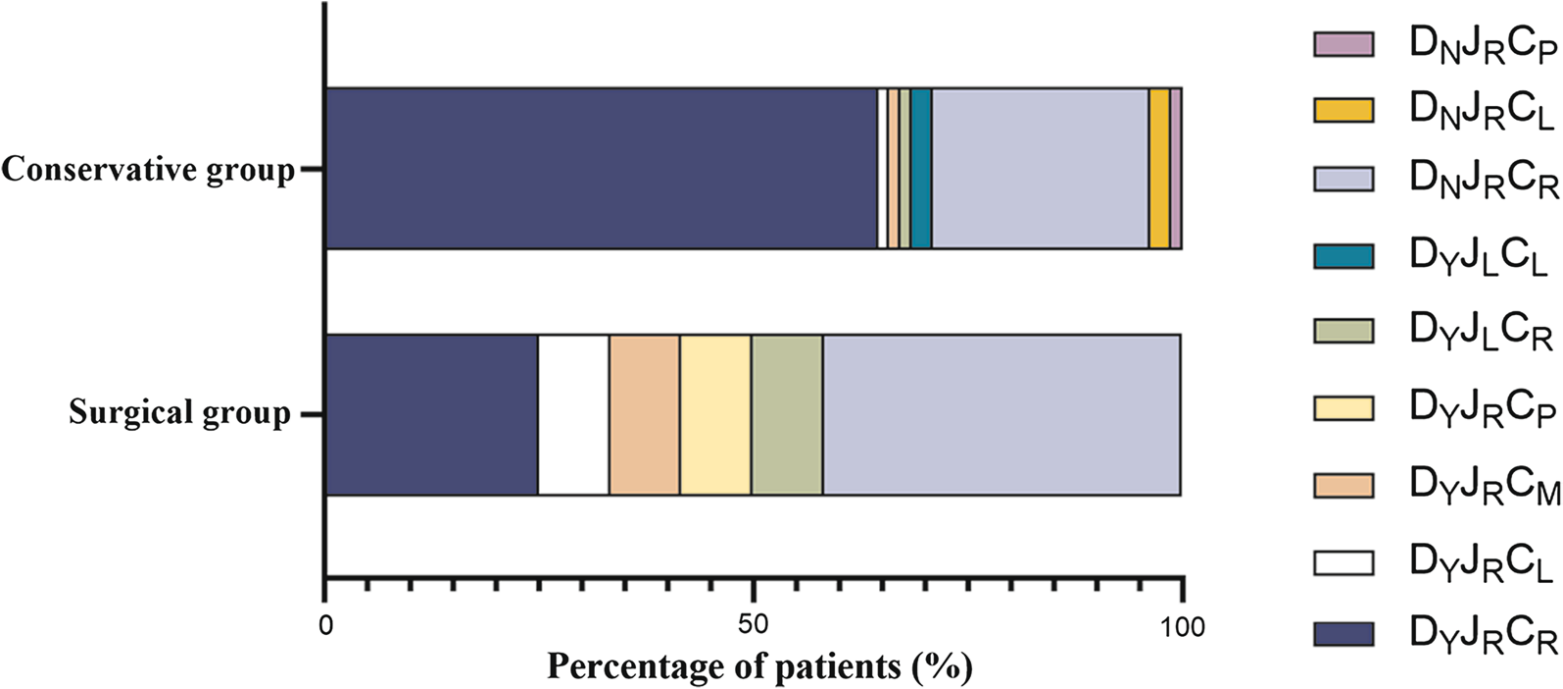
Distribution of CT classification of patients in the surgical and conservative groups

There was no significant difference in gender distribution or mean age between the two groups (*p* = 0.76 and 0.89, respectively). Abdominal pain was a common symptom in both groups (*p* = 0.45). The incidence of intestinal obstruction and volvulus was significantly higher in the surgically managed group compared to the conservatively managed group (*p* = 0.002 and 0.046, respectively). Of the seven patients who developed volvulus, none had SMA/SMV inversion, but all demonstrated SMV angulation. Besides, the “whirlpool sign” was observed in six of them, and volvulus was confirmed intraoperatively in the remaining one patient. There was no significant difference in the distribution of symptom durations between the two groups (*p* = 0.36), but half of the patients in the surgical group had chronic onset, while 24.1% of the patients in the conservative group showed acute symptoms (Additional file 1: Fig. S2). The CT classification of the two groups was also compared as shown in Fig. 5, which showed that the conservative group was predominantly D_Y_J_R_C_R_ (64.5%, 51/79) (type 4a-b), while the surgical group was predominantly D_N_J_R_C_R_ (41.7%, 5/12) (type 1a–b). There was a statistically significant difference in the CT classification of the two groups (*p* = 0.016) with the conservatively managed group demonstrating more heterogeneity. In addition, no abnormalities in the SMA/SMV relationship were observed in the surgically managed group. No statistical differences between the two groups (*p* = 0.75) were observed with respect to mesenteric vessel orientation.

## Discussion

In this study, intestinal variations of malrotation were categorized into ten groups based on three common anatomical landmarks (duodenum, jejunum, and cecum) on CT images (Fig. 2), a classification scheme easily adaptable to clinical practice and easily understandable by clinicians. Furthermore, CT results from these patients were further analyzed with respect to their clinical manifestations and treatments received in an effort to facilitate identification of high-risk patients by their CT presentation, thus enabling individualized treatment and follow-ups. To our knowledge, this is the largest clinical and radiological study of adult intestinal malrotation to date.

Compared with infants and children, the existence of adult intestinal malrotation has been historically ignored due to the lack of characteristic clinical features and is often described as an unexpected discovery in the literature [19, 20]. However, the current study confirms that malrotation is not only a condition to be recognized by pediatricians, but also need to be taken into account by gastroenterologists and surgeons when diagnosing adult patients. In 332 adult patients, this abnormality occurred in any age (mean age 51.0 ± 15.3 years, range 18–97) and was twice as common in men (male/female: 1.91:1) a finding significantly different from previous studies (Durkin et al. 0.43:1 [5], Nehra et al. 0.78:1 [6], and Anand et al. 1:1 [4]).

Imaging studies of intestinal malrotation are infrequent, and studies with large sample sizes are even rarer. Long et al. [13] performed a typology of intestinal malrotation by analyzing the intestinal distribution on pediatric UGI series, after which Fay et al. [21] utilized radiation-free MRI for further study of malrotation in children, confirming its diagnostic reliability. However, the value of CT has traditionally been underestimated. Although the current gold standard for the diagnosis remains UGI series, a number of authors including Zissin et al. [19] have highlighted the unique advantages of abdominal CT in the diagnosis of malrotation in adults. In Yang et al. [18], CT manifestations associated with an increased incidence of complications were described; however, that work only included 14 patients. Based on the analysis of 332 patients in the present study, the advantages of CT in detection of asymptomatic malrotation are further confirmed. Moreover, the D_N_J_R_C_R_ subtype (1a–b) was most common in patients undergoing surgery, whereas the D_Y_J_R_C_R_ subtype (4a–b) was most common in those managed conservatively. These findings may help clinicians detect more insidious forms of malrotation, guiding subsequent treatment and follow-up.

The current findings suggest that the presence of the jejunum in the right abdomen is a consistent feature of intestinal malrotation, present in 98.2% (326/332) of patients. Cecal ectopia was found in 12% (40/332) of patients with 29 cases located on the left, 7 in the pelvis, and 4 in the middle of the abdominal cavity, a more common finding than in Yang et al. (1/14) [18]. Cecal ectopia has been described in several case reports [22–25], and patients may develop atypical abdominal pain in the presence of concurrent appendicitis, requiring prompt identification. Sonomura et al. emphasized the high diagnostic value of coronal CT in acute appendicitis with intestinal malrotation [25] which seems reasonable considering the uncertainty regarding the location of the appendix in patients with malrotation. Although an abnormal SMA/SMV relationship was only observed in 8.4% (28/332) of patients, 20 cases were asymptomatic (Additional file 1: Table S1). Abnormal SMV angulation was observed in 98.7% of patients with an otherwise normal SMA/SMV relationship. Anatomic inversion between SMA and SMV found on CT and US has been proposed as a feature of intestinal malrotation [26], but this abnormal anatomical relationship has been shown to be inadequate in the diagnosis of isolated malrotation (observed in only 28% of patients with malrotation) [16] and can also be observed in normal people [27]. Therefore, in the diagnosis of malrotation, not only the relative position of the SMA and SMV should be noted, but also abnormal angulation of the SMV.

Furthermore, 91 patients with detailed clinical information and treatment history were analyzed. Not surprisingly the incidence of both intestinal obstruction and volvulus were significantly higher in the surgically managed group (*p* = 0.002 and 0.046, respectively). In addition, although the mean age of the two groups was similar, the conservatively managed group encompassed a wider age range. Older patients in particular were more likely to be treated conservatively, consistent with previous findings [28]. This may be attributable to older patients, in general, being poorer surgical candidates. Interestingly, although half of the patients in the surgical group had a short duration of illness, 33.3% (4/12) also had a duration of illness longer than one month and two of them even longer than one year. In addition patients in the conservative treatment group had more symptoms compared to the surgical group. This could be due to two factors: The conservative group had a larger number of cases, and patients in it generally had a longer duration of disease, which makes them more likely to have various gastrointestinal symptoms. Although further analysis showed that this difference was not statistically significant, such factors should still be taken into account.

Significant differences in CT classification were observed between the two groups. D_Y_J_R_C_R_ subtype (4a-b) was present in up to 64.5% of the conservatively managed group but only in 25% of the surgically managed group (*p* = 0.013). In Yang et al., volvulus occurred more frequently in patients with partial rotation of the duodenum and colon [18], consistent with the current results. However, although six of the seven patients [D_Y_J_R_C_R_ (Fig. 2, type 4a–b): 5, D_Y_J_R_C_M_ (Fig. 2, type 6): 1, and D_N_J_R_C_R_ (Fig. 2, type 1a): 1] who developed volvulus exhibited duodenal partial rotation, four of them with D_Y_J_R_C_R_ subtype had less severe volvulus that improved with conservative treatment. These results suggest that patients with a partially rotated duodenum are less likely to require surgery when the cecum is in a normal position. But the small number of patients with volvulus in both studies limits the strength of these findings. Moreover, although none of the patients with volvulus had an abnormality in the relative position of SMV and SMA, we observed SMV angulation in all of their CT images, and in addition, most of them (6/7) showed the typical “whirlpool sign.” Previous studies have confirmed the sensitivity of the “whirlpool sign” in CT and US images in the diagnosis of volvulus [12, 29], and the current study also supports this.

Another unexpected finding in the current study was that the proportion of asymptomatic patients was as high as 56.6% (188/332), much higher than the 17% (14/82, *p* < 0.001) in a previous study [6]. Schey et al. [30] have suggested that patients with duodenal nonrotation are more often asymptomatic, which is further confirmed by the radiological study by Zissin et al. [19]. However, in the current study, only 19.1% (36/188) of asymptomatic patients exhibited duodenal nonrotation, and further, such patients tended to have a variety of other intestinal abnormalities (Additional file 1: Table S1). In addition, the “whirlpool sign” was observed in four asymptomatic patients, which mainly showed mild rotation of the vessels and mesentery, without intestinal torsion, explaining their lack of symptoms upon presentation. In fact, many asymptomatic patients diagnosed incidentally can recall bouts of abdominal discomfort potentially related to their malrotation if their past symptoms are thoroughly interrogated [2, 31]. In the present study, the majority of asymptomatic cases, except for some diagnosed incidentally during routine physical examinations, were detected as a result of an examination or treatment for coexisting disease, and even if there were symptoms that might be associated with malrotation, it was difficult to definitely attribute them as such. In another CT study [19], the proportion of asymptomatic patients also reached 94.4% (17/18). However, these patients likewise could have been only temporarily asymptomatic or only had only mild symptoms that were insufficiently severe to report. Yet, these individuals would still potentially be at risk for future volvulus. The incidence of volvulus in the current study was only 2.1% (7/332), much lower than the 12% in Nehra et al. (*n* = 82, *p* < 0.001) [6]. The increasing adoption of abdominal CT may improve reader confidence in identifying asymptomatic cases of malrotation, thus reducing the proportion found in the setting of volvulus.

**Fig. 6:**
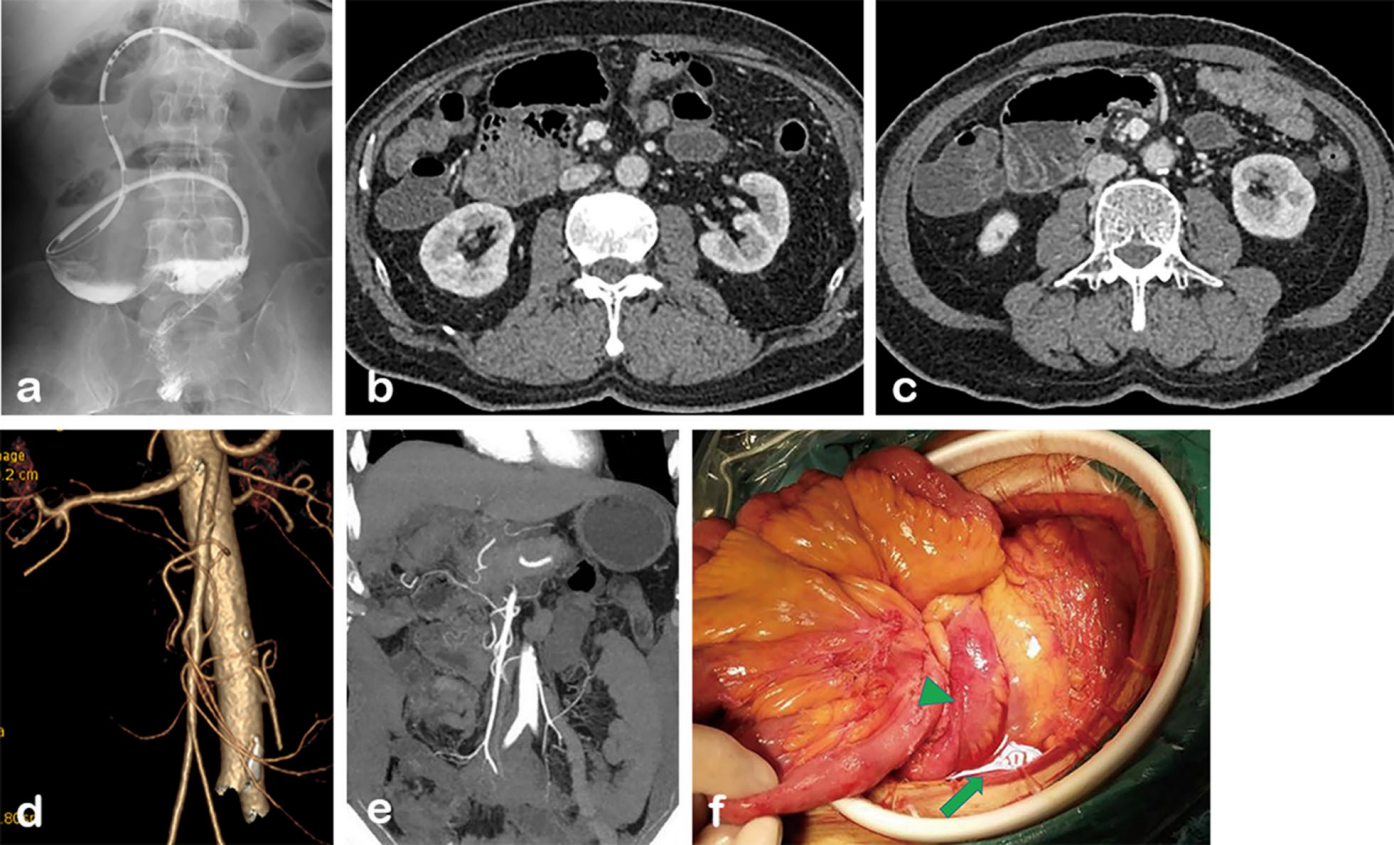
A 64-year-old man developed volvulus after radical resection of rectal cancer (D_N_J_R_C_R_, type 1a). **a** Endoscopic catheterization: After passing through the duodenum, which was located to the right of the midline, the catheter was unable to pass smoothly into the misplaced obstructed proximal jejunum. **b**, **c** CT showed that the duodenum and jejunum were located in the right upper abdomen. **d**, **e** CTA shows SMA and its branches. 6f: Ladd’s bands were found during surgery (arrow). The terminal ileum was visualized (arrowhead)

Currently, the Ladd’s procedure is a widely accepted standard treatment for intestinal malrotation, and some studies have emphasized that the Ladd’s procedure should be routinely used in patients with intestinal malrotation, regardless of age [32, 33]. However, in the present study, the rate of surgery was only 13.2% (12/91), much lower than previously reported (Additional file 1: Fig. S3), and of the 12 patients who underwent surgery, only three were treated with Ladd’s procedure (Fig. 6), which seems to suggest an overall inadequate understanding of the disease and its treatment. In fact, although surgery is sometimes necessary, it also should be pursued cautiously. Compared to infants and children, adult patients are more likely to have major postoperative complications and have a higher reoperation rate [5, 34]. In addition, some patients with intestinal necrosis will undergo partial small bowel resection. In this setting, inadequate identification or evaluation of malrotation preoperatively may lead to a more complicated surgery and adverse outcomes. Hsu et al. have reported on short bowel syndrome caused by undiagnosed intestinal malrotation resulting from inappropriate surgical maneuvers and emphasize that CT should be utilized to adequately evaluate each patient preoperatively, especially for procedures with limited field of view, such as laparoscopic and Da Vinci robotic surgery [35]. Given the increasing popularity of minimally invasive surgery, number of asymptomatic patients with malrotation, and various malrotation subtypes, adequate scrutiny of the proximal bowel and mesenteric vessels on preoperative CT is increasingly important.

The current study has several limitations. First of all, although 332 patients were included in this study, detailed clinical data could only be examined in 91. However, this remains the largest study of adult malrotation to date. Secondly, a large number of patients were asymptomatic; although, since this was a retrospective study spanning 7 years and most patients had comorbidities, this was difficult to truly validate. In addition, we must emphasize that the CT classification we proposed is a summary description of common anatomical features and the anatomy of the same subtype may also vary, which requires a specific analysis by the radiologist. Finally, follow-up data could not be reliably obtained, preventing assessment of the effects of various treatments. Long-term follow-up of such patients will be crucial in future studies.

In summary, adult intestinal malrotation is much more common than typically considered. CT not only allows the detection of a greater number of asymptomatic patients but also facilitates diagnostic precision enabling classification of malrotation into subtypes.

## Supplementary Information

**Additional file 1.** **Supplementary Methods:** Diagnostic criteria for intestinal malrotation. **Table S1:** CT findings of 188 asymptomatic patients. **Fig. S1:** Flowchart for inclusion of patients. **Fig. S2:** Duration of symptoms in the 91 patients for whom detailed clinical data were available. **Fig. S3:** Comparing the treatment modality of patients in our study to previous studies.

## Data Availability

The datasets used or analyzed during the current study are available from the corresponding author on reasonable request.

## Abbreviations

SMA: Superior mesenteric artery
SMV: Superior mesenteric vein
UGI: Upper gastrointestinal
