# Supplementary material for: CT facilitates improved diagnosis of adult intestinal malrotation: a 7-year retrospective study based on 332 cases
Source: Insights Imaging. 2021 Apr 30;12:58. doi: 10.1186/s13244-021-00999-3 (PMC8087751; doi:10.1186/s13244-021-00999-3)

**Additional file 1**

All patients without surgical confirmation were diagnosed by consensus, a procedure that referenced the definition of malrotation from the literature: Any deviation from the normal course of duodenum is termed malrotation or nonrotation (the normal duodenum should cross the midline after descending and then ascend to the level of the pylorus) [S1].

The imaging data of each patient was evaluated by three radiologists, and agreement of at least two were required to achieve the diagnosis of intestinal malrotation. Abnormalities of the duodenum mainly included: a duodenum completely confined to the right side of the abdomen, not crossing midline; duodenum crossing midline not ascending to the level of the pylorus to form a normal duodenojejunal flexure; twisted duodenum, mesentery, and vessels forming a “whirlpool sign” in the setting of volvulus.

**Reference:**

S1: Terry B, Intestinal malrotation in adults. UpToDate. <https://www.uptodate.com/contents/intestinal-malrotation-in-adults?search=intestinal%20malrotation&source=search_result&selectedTitle=2~44&usage_type=default&display_rank=2.> (Accessed on Nov 30, 2020).

**Table S1 CT findings of 188 asymptomatic patients**

|  | Total (n=188)  No. (%) |
| --- | --- |
| **CT classification** |  |
| Duodenal partial rotation | 151 (80.3) |
| D_Y_J_R_C_R_ | 139 |
| D_Y_J_R_C_L_ | 9 |
| D_Y_J_R_C_M_ | 2 |
| D_Y_J_R_C_P_ | 1 |
| Duodenal nonrotation | 36 (19.1) |
| D_N_J_R_C_R_ | 21 |
| D_N_J_R_C_L_ | 11 |
| D_N_J_R_C_P_ | 4 |
| Duodenal rotation |  |
| D_Y_J_M_C_R_ | 1(0.5) |
| **Position of SMA relative to SMV** |  |
| Left rear | 94 (50.0) |
| Left front | 41 (21.8) |
| Left | 33 (17.6) |
| Right rear | 20 (10.6) |
| **Whirlpool sign** | 4 (2.1) |

*SMA*, superior mesenteric artery; *SMV*, superior mesenteric vein.

**Fig. S1** Flowchart for inclusion of patients


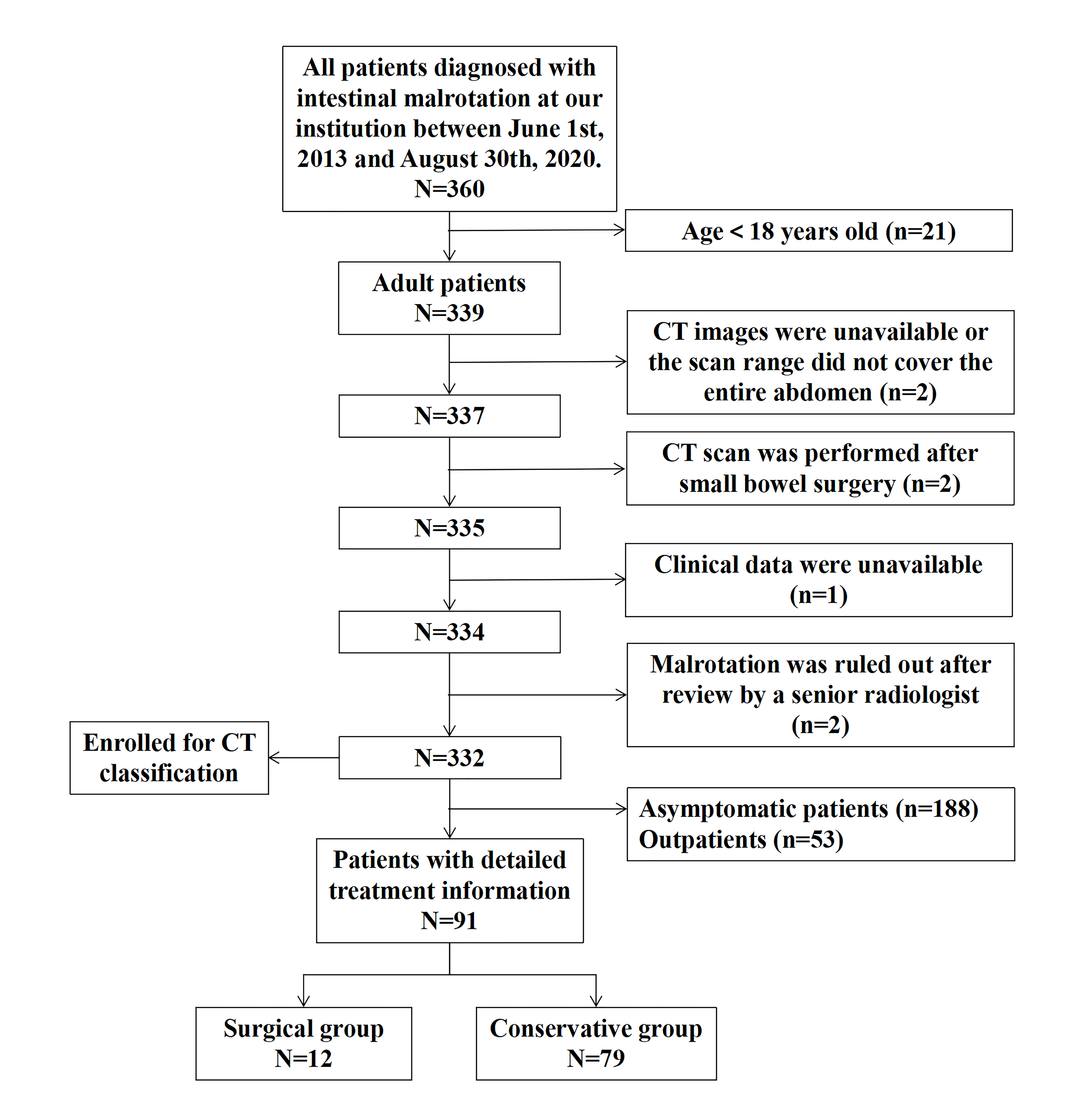


**Fig. S2** Duration of symptoms in the 91 patients for whom detailed clinical data were available.


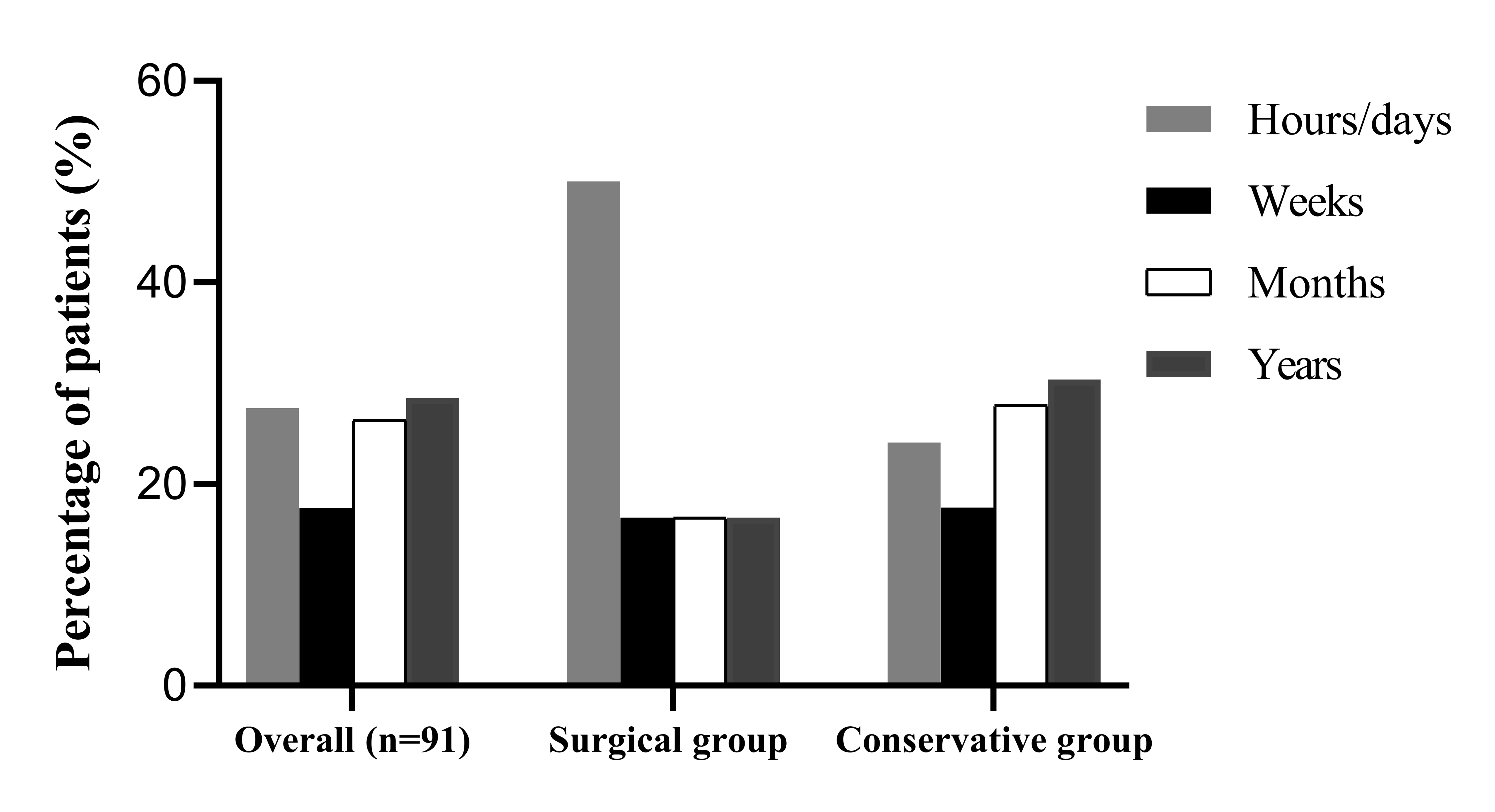


**Fig. S3** Comparing the treatment modality of patients in our study to previous studies.

The surgery rate in our patients was only 13.2%, which is significantly lower than previous studies (Durkin et al.’s 90%, Nehra et al.’s 61%, and Anand et al.’s 100%; *p*＜.001).


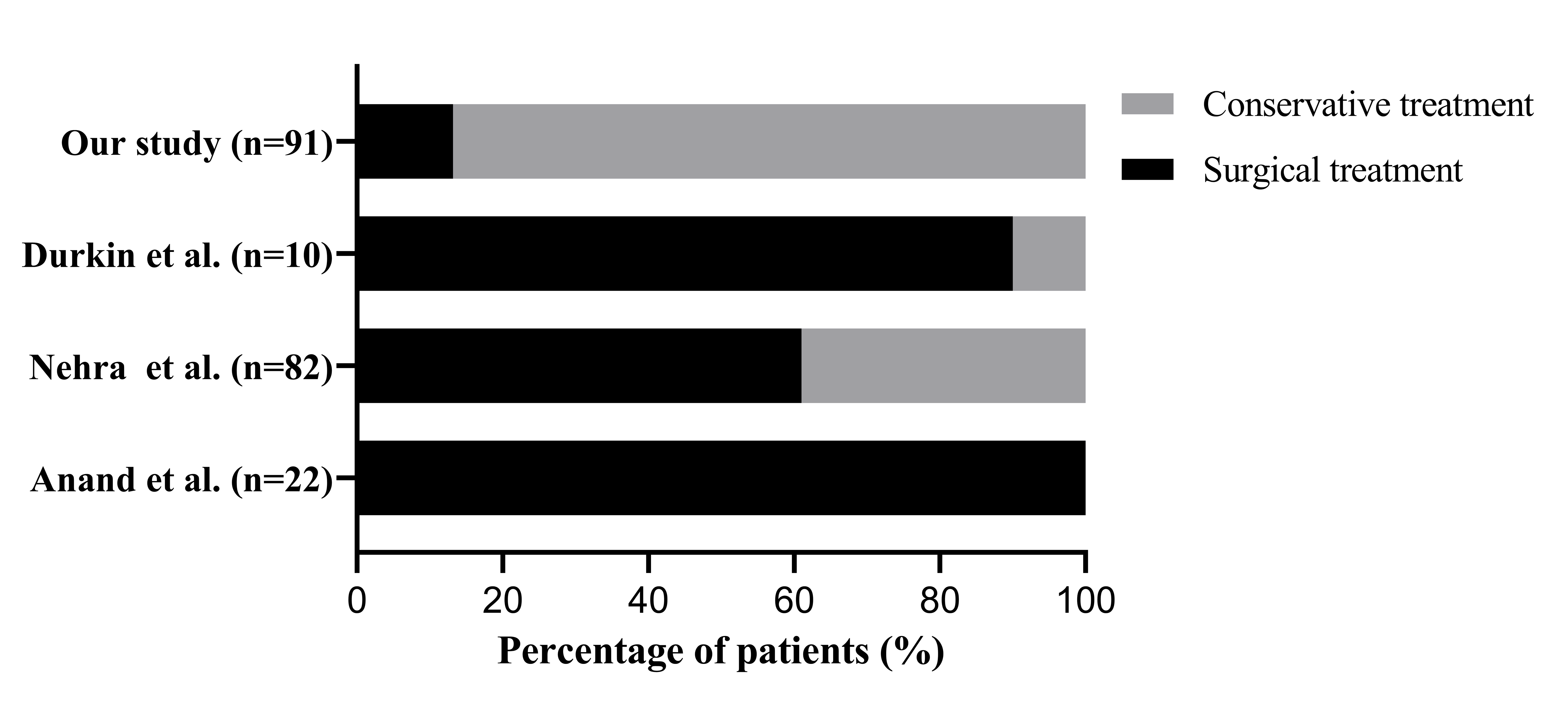

Supplement: Supplementary file 1 — Additional file 1. Supplementary Methods: Diagnostic criteria for intestinal malrotation. Table S1: CT findings of 188 asymptomatic patients. Fig. S1: Flowchart for inclusion of patients. Fig. S2: Duration of symptoms in the 91 patients for whom detailed clinical data were available. Fig. S3: Comparing the treatment modality of patients in our study to previous studies. [file 13244_2021_999_MOESM1_ESM.docx]
